# An autophagy-related long non-coding RNA prognostic model and related immune research for female breast cancer

**DOI:** 10.3389/fonc.2022.929240

**Published:** 2022-12-15

**Authors:** Jiafeng Chen, Xinrong Li, Shuixin Yan, Jiadi Li, Yuxin Zhou, Minhua Wu, Jinhua Ding, Jiahui Yang, Yijie Yuan, Ye Zhu, Weizhu Wu

**Affiliations:** ^1^ Department of Thyroid and Breast surgery, The Affiliated Lihuili Hospital, Ningbo University, Ningbo, China; ^2^ School of Medicine, Ningbo University, Ningbo, China

**Keywords:** breast cancer, long non-coding RNAs, tumor immune microenvironment, prognostic model, autophagy, survival

## Abstract

**Introduction:**

Breast cancer (BRCA) is the most common malignancy among women worldwide. It was widely accepted that autophagy and the tumor immune microenvironment play an important role in the biological process of BRCA. Long non-coding RNAs (lncRNAs), as vital regulatory molecules, are involved in the occurrence and development of BRCA. The aim of this study was to assess the prognosis of BRCA by constructing an autophagy-related lncRNA (ARlncRNA) prognostic model and to provide individualized guidance for the treatment of BRCA.

**Methods:**

The clinical data and transcriptome data of patients with BRCA were acquired from the Cancer Genome Atlas database (TCGA), and autophagy-related genes were obtained from the human autophagy database (HADb). ARlncRNAs were identified by conducting co‑expression analysis. Univariate and multivariate Cox regression analysis were performed to construct an ARlncRNA prognostic model. The prognostic model was evaluated by Kaplan–Meier survival analysis, plotting risk curve, Independent prognostic analysis, clinical correlation analysis and plotting ROC curves. Finally, the tumor immune microenvironment of the prognostic model was studied.

**Results:**

10 ARlncRNAs(*AC090912.1, LINC01871, AL358472.3, AL122010.1, SEMA3B-AS1, BAIAP2-DT, MAPT-AS1, DNAH10OS, AC015819.1, AC090198.1*) were included in the model. Kaplan–Meier survival analysis of the prognostic model showed that the overall survival(OS) of the low-risk group was significantly better than that of the high-risk group (p< 0.001). Multivariate Cox regression analyses suggested that the prognostic model was an independent prognostic factor for BRCA (HR = 1.788, CI = 1.534–2.084, p < 0.001). ROCs of 1-, 3- and 5-year survival revealed that the AUC values of the prognostic model were all > 0.7, with values of 0.779, 0.746, and 0.731, respectively. In addition, Gene Set Enrichment Analysis (GSEA) suggested that several tumor-related pathways were enriched in the high-risk group, while several immune‑related pathways were enriched in the low-risk group. Patients in the low-risk group had higher immune scores and their immune cells and immune pathways were more active. Patients in the low-risk group had higher PD-1 and CTLA-4 levels and received more benefits from immune checkpoint inhibitors (ICIs) therapy.

**Discussion:**

The ARlncRNA prognostic model showed good performance in predicting the prognosis of patients with BRCA and is of great significance to guide the individualized treatment of these patients.

## 1 Introduction

Breast cancer (BRCA) is both the most common malignant tumor and the most common cause of cancer death in women worldwide. According to available data, the incidence and mortality rates of BRCA are increasing in low-resource countries (1). At present, BRCA treatment is still based on surgery, combined with other adjuvant therapies, such as chemotherapy, radiotherapy, immunotherapy, endocrine therapy, and targeted therapy (2–5). Some types of BRCA, especially triple-negative breast cancer (TNBC), have a poor prognosis (5). Therefore, identifying biomarkers that can predict the prognosis of BRCA, establishing prognostic models, and searching for new therapeutic sites are considered to be of high clinical importance.

Long non-coding RNAs (lncRNAs) are defined as a series of non-coding RNAs that contain more than 200 bases without protein-coding function (6), which have crucial biological functions. Some lncRNAs are abnormally expressed in tumor tissues, and their abnormal expression is closely related to tumor occurrence, metastasis, tumor stage, and the survival rates. For example, lncRNAs *PCA3*, *PCGEM1*, and *PCAT-1* are highly expressed in prostate cancer, and the expression level of *KIAA0125* correlated negatively with the prognosis of acute myeloid leukemia (AML) (7). The expression of *PVT1* was significantly increased in gastric cancer, while that of *ZFAS1* showed the opposite pattern (8). These observations reveal the potential for finding targets for the diagnosis, treatment, and prognosis of cancers (9, 10).

**Figure 1:**
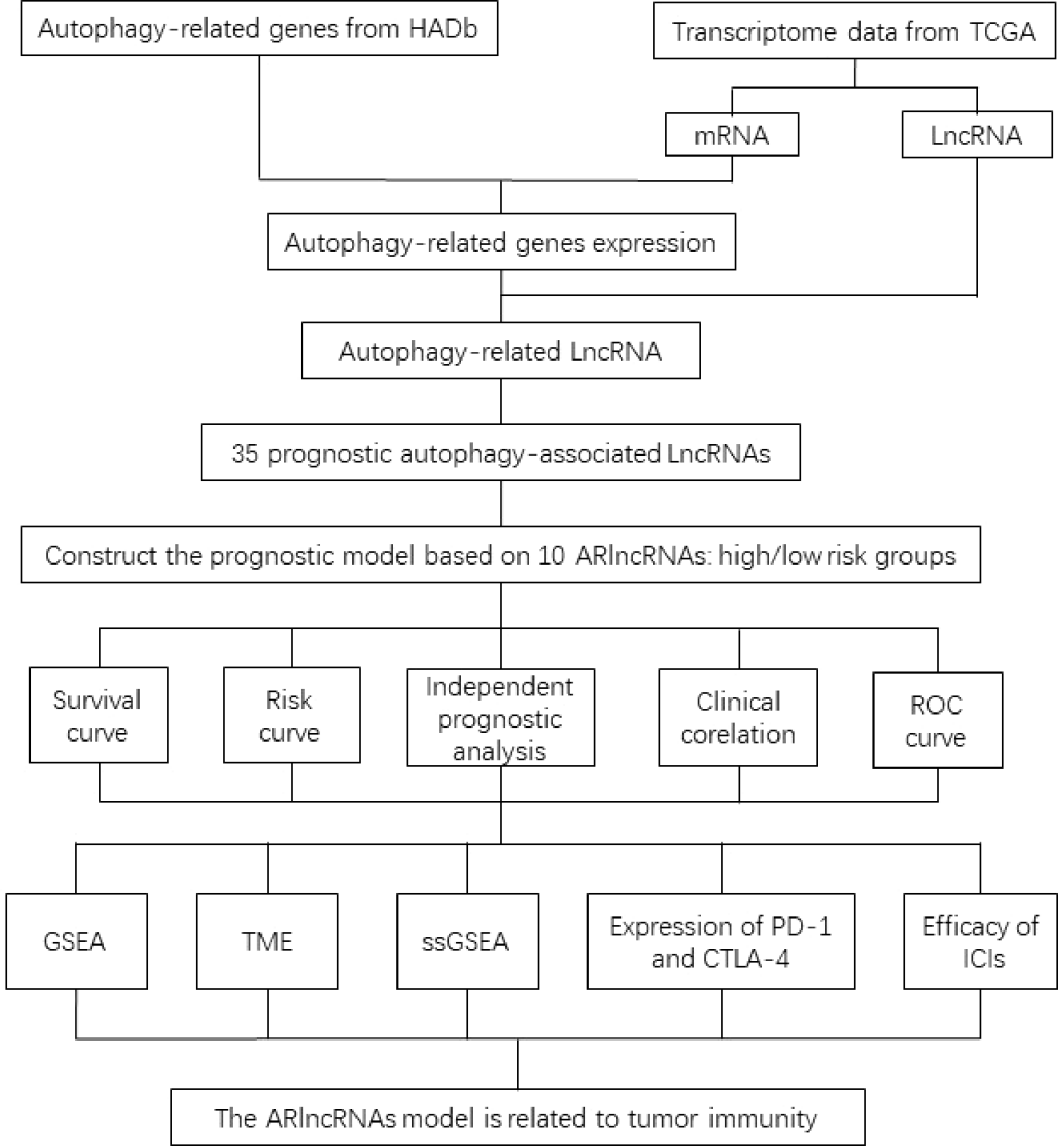
Flow chart of the construction of the prognostic mode of ARlncRNAs in breast cancer.

Autophagy is a physiological process in which substances are recycled through lysosome degradation to maintain cell homeostasis (11, 12). Mutations in autophagy-related genes have been associated with a variety of diseases (13). Upregulation of autophagy can promote the occurrence, development, and drug resistance of cancer (14, 15). A series of studies confirmed that autophagy plays a crucial role in the biological behavior of BRCA (16). In addition, the correlation between autophagy and the tumor immune microenvironment (TME) has been reported in several studies. For example, Kuo et al. (17) found that autophagy can regulate tumor-associated macrophages (TAMs) in the TME, thereby affecting cancer progression. Jiang et al. (18) found that autophagy established a connection with the TME in three aspects and suggested that it was expected to improve the effectiveness of immunotherapy by regulating autophagy. In this study, we constructed a prognostic model of BRCA based on 10 autophagy-related lncRNAs (ARlncRNAs). In addition, we analyzed the differences in the TME in the high-/low-risk groups and evaluated the effectiveness of immune checkpoint inhibitors (ICIs) in the two groups; the technical route is shown in **Figure 1**. The results of the present study provide new guidance for individualized treatment of BRCA.

## 2 Methods and materials

### 2.1 Data acquisition and processing

The clinical data and transcriptome data of patients with BRCA were acquired from the Cancer Genome Atlas database (TCGA, https://cancergenome.nih.gov/), and autophagy-related genes were obtained from the human autophagy database (HADb, http://www.autophagy.lu/). The expression matrices of lncRNAs and mRNAs were obtained from transcriptome data, and the expression matrix of autophagy-related genes was extracted from the expression matrix of the mRNA. Finally, ARlncRNAs were identified through the construction of an autophagy-related mRNA–lncRNA co-expression network using Pearson correlation analysis according to the following criteria: |correlation coefficient| > 0.3 and p< 0.001, using the limma package (19). In this study, the clinical data of 1,041 women with breast cancer were analyzed, and 862 patients were included for subsequent data analysis.

### 2.2 Construction of prognostic model

We performed Kaplan–Meier survival analysis combined with univariate Cox analysis; the key ARlncRNAs that correlated significantly with overall survival (OS) and their expression levels in each sample with BRCA were screened out, according to the standard of log-rank p< 0.05 and Cox p< 0.05, respectively.

Kaplan–Meier approach was used to estimate the survival rate at all time points of death. The survival rate was calculated by the following formula.


$$Surv\text{i}val\;rat\text{e}=p_{1}*p_{2}*p_{3}*\ldots *p_{i}$$

That is, the survival rate at a certain time is equal to the product of the survival probability of the nodes where each death event occurs before.

The selected ARlncRNAs were then subjected to multivariate Cox regression analysis and optimized according to the Akaike information criterion (AIC = 1,531.72) to construct the optimal risk score prognostic model by running Survival R packages. Finally, the risk score of each patient with BRCA was acquired using the following formula.


$$Risk\;score=\sum_{i=1}^{10}coef\left(lncRNAi\right)*expr\;\left(lncRNAi\right)$$

where coef (lncRNAi) represents the regression coefficient of the corresponding ARlncRNA correlated with survival, and expr (lncRNAi) represents the expression of the ARlncRNA. Patients with BRCA in the TCGA were divided into different groups according to their median risk score.

### 2.3 Evaluation of the prognostic model

Kaplan–Meier (KM) survival analysis was performed to compare the OS between different levels of ARlncRNAs expression in the prognostic model and the OS between different risk groups, using the R survival package. In addition, according to the risk score of each patient, survival status and the expression levels of the 10 ARlncRNAs in the prognostic model, boxplot, risk curve, survival status scatter plot, and risk heat map were generated, respectively. To confirm whether the prognostic risk model is an independent risk factor for patients with BRCA, univariate and multivariate Cox regression analyses were conducted, and forest maps were generated. To confirm the correlation between risk scores and clinicopathological features, we divided age, stage, T (tumor), M (metastasis), and N (node) into two groups and performed T-tests to evaluate whether there were significant differences in risk scores between the above groups. Moreover, we drew a receiver operating characteristic (ROC) curve and calculated the area under the curve (AUC) using the survivalROC package to compare the predictive value between prognostic risk model, age, stage, and TNM status.

### 2.4 Gene set enrichment analysis in the prognostic model

To identify differentially expressed genes between the different risk groups, we used the limma package to perform differential expression analysis, with log fold change (FC) > 1 and false discovery frate (FDR)< 0.05 as screening criteria. Gene Ontology (GO) functional enrichment analysis was used to identify the GO terms most likely associated with the differentially expressed genes and to determine the enrichment degree of the differentially expressed genes in functional phenotypes (p< 0.05). In addition, Kyoto Encyclopedia of Genes and Genomes (KEGG) pathway enrichment analysis was performed by using Gene Set Enrichment Analysis (GSEA) 4.1.0 software (p< 0.05 and q< 0.25). Meanwhile, we performed GO and KEGG enrichment analysis for autophagy-related genes(ARGs) co-expressed with 10 ARlncRNAs in this model.

### 2.5 Correlation between tumor immune microenvironment and the prognostic model

To explore the differences in the content of immune cells and stromal cells between the high-/low-risk groups, limma and the estimate package in the R software were performed, and the results were represented by ImmuneScore, StromalScore, and ESTIMATEScore (= StromalScore + ImmuneScore). Wilcoxon rank sum tests were conducted to assess whether there were differences in ImmuneScore, StromalScore, and ESTIMATEScore between the two groups, and then, the results were visualized. Single-sample GSEA (ssGSEA) was performed to score the set of immune-related genes in each BRCA sample to evaluate the degree of enrichment of immune-related genes, using limma, GSVA, and GSEABase packages.

### 2.6 Efficacy evaluation of immunotherapy in the prognostic model

Immunotherapy is changing the treatment strategy for a variety of solid tumors, and studies have shown that ICIs have therapeutic activity in some patients with BRCA (3). Programmed cell death 1 (PD-1) and cytotoxic T-lymphocyte associated protein 4 (CTLA-4) are target molecules for ICIs; therefore, differential analysis of PD-1 and CTLA-4 expression levels in the prognostic model was conducted using Wilcoxon rank sum tests to explore whether there were statistical differences in PD-1 and CTLA-4 expression levels between the high-/low-risk groups, which generated two box plots. The immunophenoscore (IPS), data representing the efficacy of immunotherapy in patients with BRCA, was obtained from The Cancer Immunome Atlas (TCIA, https://tcia.at/home). Wilcoxon rank sum tests were used to calculate the difference in efficacy of anti-PD1 and anti-CTLA4 therapy between the low- and high-risk groups, and three violin plots were generated.

## 3 Results

### 3.1 Identification of ARlncRNAs and construction of the prognostic model

**Figure 2:**
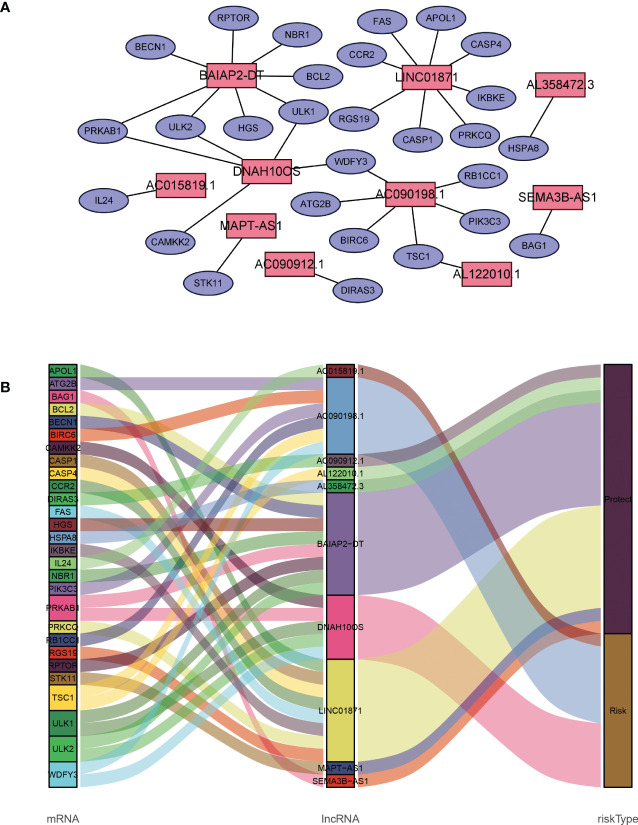
Description of the regulatory relationship between ARlncRNAs and ARGs in BRCA. **(A)** Co-expression network of ARlncRNAs and ARGs created using Cytoscape 3.8.2. **(B)** Co-expression of ARGs and ARlncRNAs and the prognostic value of 10 ARlncRNAs.

A total of 19,658 mRNAs and 14,142 lncRNAs were identified from the transcriptome matrix, which was downloaded from the TCGA. There 232 autophagy-related genes (**Supplementary File 1**) that were obtained from HADb, and 1,272 ARlncRNAs were identified by conducting co-expression analysis (**Supplementary File 2**). Combined with the KM survival analysis and univariate Cox analysis, 35 ARlncRNAs (**Table 1**) associated with survival were screened. Ultimately, 10 ARlncRNAs (**Table 2**) were identified by multivariate Cox regression analysis and included in the model. Each patient with BRCA was assigned to different groups based on their median risk score. Finally, the above mentioned 10 ARlncRNAs and their co-expressed mRNAs were used to construct co-expression network (**Figure 2A**) and generated a Sankey diagram (**Figure 2B**).

**Table 1:** Thirty-five ARlncRNAs significantly correlated with OS by univariate Cox analysis combined with Kaplan–Meier survival analysis.

ID	KM	p-value	HR
PCED1B-AS1	0.004095126	0.022446	0.890652
LINC01235	0.011893223	0.015454	1.011234
TNFRSF14-AS1	0.001055979	0.003987	0.559176
AC004067.1	0.022394956	0.03188	0.660425
AC136475.2	0.011996657	0.040302	0.846127
Z68871.1	0.010352616	0.015365	1.30539
AL138724.1	0.017640288	0.03187	0.66958
LINC01614	0.029749288	0.004383	1.022387
USP30-AS1	0.006135972	0.006233	0.778554
SH3BP5-AS1	0.012297225	0.041751	0.811188
AC139768.1	0.004720735	0.015916	0.687708
AC090912.1	0.038089601	0.00896	0.500986
NIFK-AS1	0.003598391	0.009854	0.680715
AC004585.1	0.019317112	0.0382	0.822731
FLJ42351	0.012980886	0.004264	0.504715
DNAH10OS	0.021695283	0.034623	1.150627
ST7-AS1	0.008435878	0.003417	0.567933
AC107464.3	0.003041757	0.017699	0.836235
LINC01871	0.016174766	0.000602	0.794068
AL358472.3	0.042200507	0.010851	0.751026
AL122010.1	0.004815823	0.000884	0.724019
SEMA3B-AS1	0.022572035	0.013924	0.925988
STAG3L5P-PVRIG2P-PILRB	0.013239726	0.034014	0.706408
AC015819.1	0.016608965	0.048489	0.777723
AC005034.5	0.029715848	0.000471	1.341049
AC234582.1	0.001801122	0.024824	0.745494
OTUD6B-AS1	0.037794728	0.00578	1.078476
AC121761.2	0.00315185	0.02591	0.613971
AC090948.3	0.041284337	0.019344	0.611236
AC061992.1	0.019349193	0.018131	0.722525
BAIAP2-DT	0.004083492	0.049533	0.950948
MAPT-AS1	0.003756485	0.001992	0.732673
AC090198.1	0.003258303	0.034444	1.076469
AL451085.2	0.011138586	0.007796	0.643652
DLG5-AS1	0.041754745	0.028418	0.850987

**Table 2:** Ten ARlncRNAs included in the ARlncRNA model and their regression coefficients and hazard ratios.

ID	Coef	HR
AC090912.1	−0.473256804650038	0.622970069023329
DNAH10OS	0.117638362645059	1.124837254634970
LINC01871	−0.340646509552672	0.711310305168542
AL358472.3	−0.235810604213364	0.789930269230725
AL122010.1	−0.228167747386301	0.795990723272214
SEMA3B-AS1	−0.054606704889089	0.946857469145977
AC015819.1	0.236477431535496	1.266778966041390
BAIAP2-DT	−0.044945144303109	0.956049925179587
MAPT-AS1	−0.310354022823099	0.733187345219075
AC090198.1	0.082275777361653	1.085755195248940

### 3.2 Evaluation of predictive efficacy of prognostic model consisting of 10 ARlncRNAs

**Figure 3:**
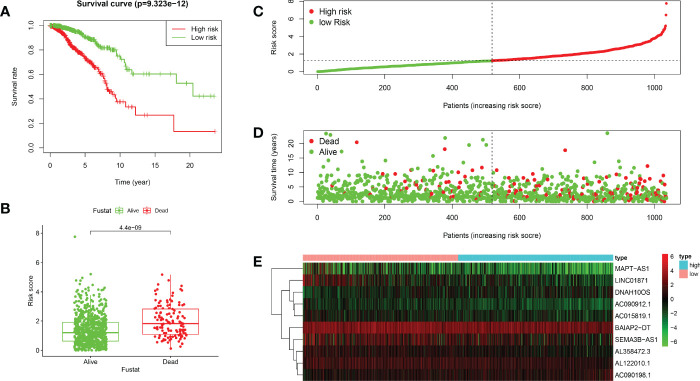
Validation of the prognostic value of the ARlncRNA model. **(A)** Survival curves for the high- and low-risk groups created using Kaplan–Meier survival analysis. **(B)** A boxplot showing that the risk scores of patients who died were significantly higher than those of patients who survived. **(C)** A risk curve based on the risk score of each patient with BRCA. **(D)** A survival scatter plot based on the survival status of each patient with BRCA. **(E)** A heatmap showing the expression levels of 10 ARlncRNAs in the low- and high-risk groups.

**Figure 4:**
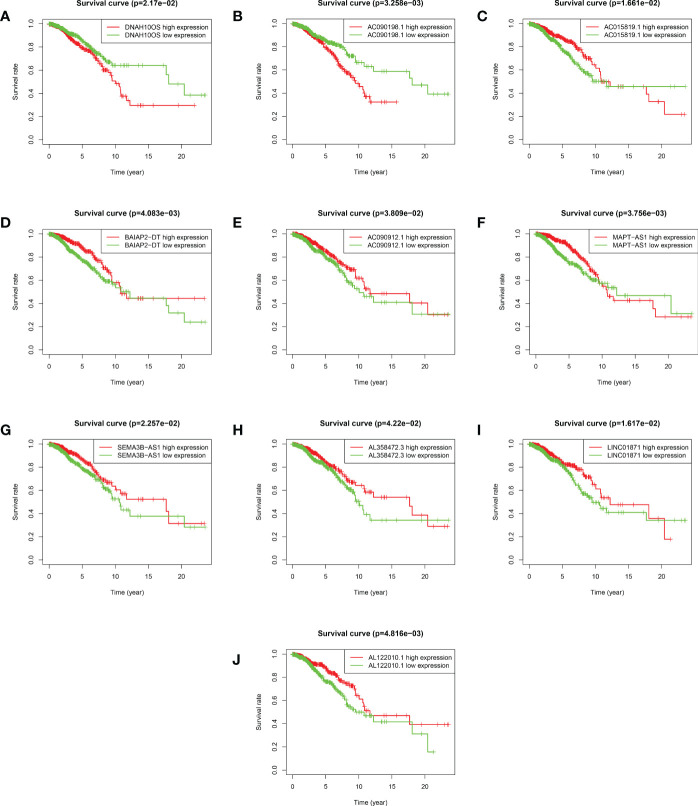
Kaplan–Meier survival analyses of 10 ARlncRNAs included in the prognosis model. **(A–J)** Survival curves of 10 ARlncRNAs and their prognostic value in BRCA.

**Figure 5:**
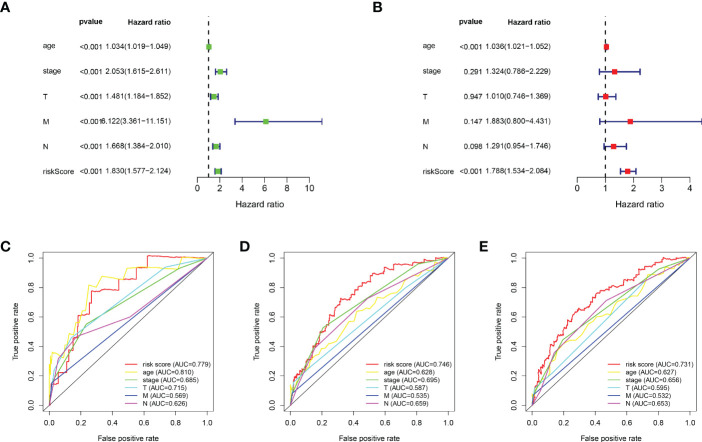
Evaluation of the predictive power of the ARlncRNA model. **(A, B)** Univariate **(A)** and multivariate **(B)** Cox regression analyses indicating that age and risk score could be independent prognostic factors for BRCA. **(C–E)** The 1- **(C)**, 3- **(D)**, and 5-year **(E)** AUC of the ARlncRNA model values were >0.7. The 3- and 5-year AUCs had the maximum values.

Based on the median value of the risk score, we divided the patients with BRCA into different risk groups, and the KM survival analysis of the model showed that the OS of the low-risk group was significantly better than that of the high-risk group (**Figure 3A**, p< 0.001). A boxplot showed that patients who died had a higher risk score than those who survived (**Figure 3B**, p< 0.001). The risk curve and survival scatter plot showed that the mortality of patients with BRCA was closely related to the risk score, and the mortality increased with increasing risk score (**Figures 3C, D**). The risk heat map showed the expression levels of these 10 ARlncRNAs in different risk groups (**Figure 3E**). The survival curves of the 10 ARlncRNAs used to construct prognostic model suggested that their expression levels were closely related to patients’ OS (**Figures 4A–J**, p< 0.05). To explore whether the prognostic model can be used as prognostic factor independent of other clinical characteristics such as age, stage, and TNM, univariate and multivariate cox regression analyses were performed, and the results indicated that two factors including age (hazard ratio (HR) = 1.036, confidence interval (CI) = 1.021–1.052, p< 0.001) and risk score (HR = 1.788, CI = 1.534–2.084, p< 0.001) could be used as independent prognostic factors (**Figures 5A, B**). To compare the predictive power of the prognostic model with that of various clinicopathological characteristics, ROCs of 1-, 3-, and 5-year survival were generated, and the results showed that the AUC values of the prognostic model were all >0.7, with values of 0.779, 0.746, and 0.731, respectively (**Figures 5C–E**). In addition, the AUC values of the prognostic model were the highest in both the 3 - and 5-year ROC. These results indicated that the ARlncRNA prognostic model has excellent predictive ability. As shown in **Table 3**, elderly patients (>65 years old), patients with advanced stage disease (stage III–IV), and patients with lymph node metastasis tended to have higher risk scores, all of which were statistically significant.

**Table 3:** The relationship between clinical features and risk scores in patients with BRCA in the ARlncRNA model.

Clinical Features		n	Risk Score			
			Mean	SD	t	p
Age						
	≤65	638	1.423	1.006	−2.575	0.010
	>65	224	1.642	1.123		
Stage						
	Stage I–II	656	1.390	1.011	−4.436	0.000
	StageIII–IV	206	1.768	1.086		
T						
	T1–2	737	1.459	1.043	-1.457	0.147
	T3–4	125	1.604	1.030		
M						
	M0	846	1.474	1.044	−1.429	0.173
	M1	16	1.792	0.879		
N						
	N0	420	1.378	1.017	−2.821	0.005
	N1–3	442	1.577	1.056		

### 3.3 GSEA in the prognostic model

**Figure 6:**
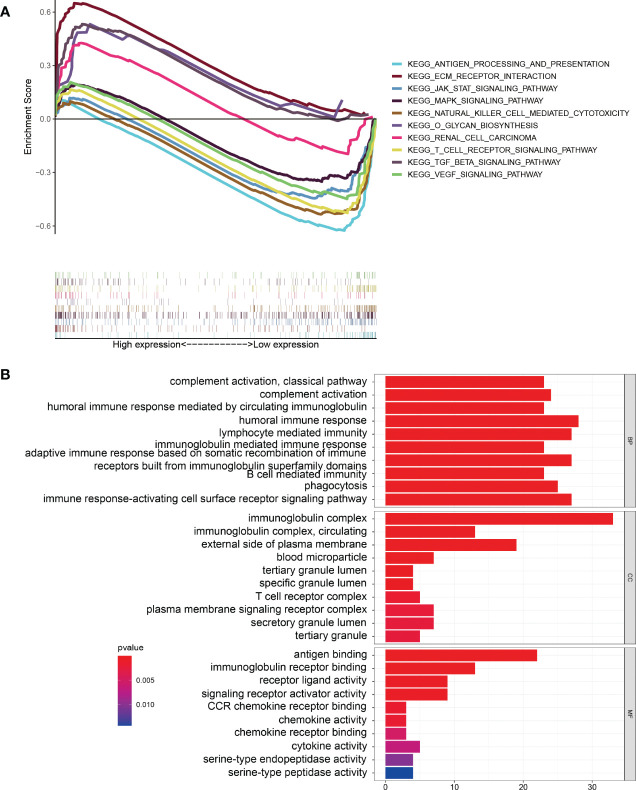
Gene Set Enrichment Analysis. **(A)** KEGG pathway enrichment analysis indicating that several immune-related pathways were enriched in the low-risk group. **(B)** GO enrichment analysis suggesting that several immune-related functions were enriched in the different groups.

KEGG pathway enrichment analysis suggested that some pathways, including extracellular matrix (ECM) receptor interaction, the transforming growth factor beta (TGF-β) signaling pathway, o-glycan biosynthesis, and renal cell carcinoma, were enriched in the high-risk group. At the same time, some immune-related pathways, such as antigen processing and presentation, natural-killer-cell mediated cytotoxicity, and T-cell receptor signaling pathway, were enriched in the low-risk group (**Figure 6A**). GO enrichment analysis indicated that tumor immune-related functions were enriched in biological process (BP), cellular component (CC), and molecular function (MF) (**Figure 6B**). In addition, the results of GO and KEGG enrichment analysis for ARGs co-expressed with 10 ARlncRNAs showed that the ARGs were heavily enriched in some autophagy-related functions and pathways. (**Supplementary File 3**).

### 3.4 Tumor immune microenvironment of the prognostic model

**Figure 7:**
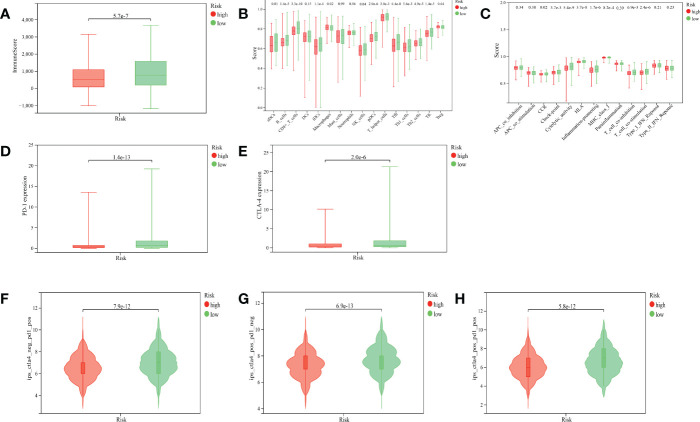
Correlation between the ARlncRNA model and tumor immunity. **(A)** A boxplot showing that patients in the low-risk group have a higher ImmuneScore. **(B)** A multi-boxplot revealing that several cells such as aDCs, B cells, CD8+ T cells, iDCs, NK cells, pDCs, T helper cells, Tfhs, Th1 cells, Th2 cells, and TILs were more active in the low-risk group, while macrophages were more active in the high-risk group. **(C)** A multi-boxplot revealing that several immune-related pathways such as CCR, checkpoint, cytolytic activity, HLA, inflammation promoting, MHC class I, T-cell co-inhibition, and T-cell co-stimulation were more active in the low-risk group. **(D, E)** Boxplots indicating that the expression levels of PD-1 **(D)** and CTLA-4 **(E)** were higher in the low-risk group. **(F, G, H)** Violin plots showing that regardless of whether it is anti-PD1 alone **(F)**, anti-CTLA4 alone **(G)**, or a combination of the two **(H)**, their efficacy in patients in the low-risk group was better than that in the high-risk group.

We found that the ImmuneScore of patients with BRCA were significantly higher in the low-risk group than in the high-risk group (**Figure 7A**, p< 0.001). The result of ssGSEA suggested that in the gene set of immune-cell-related, activated dendritic cells (aDCs), B cells, CD8+ T cells, immature dendritic cells (iDCs), natural killer (NK) cells, plasmacytoid dendritic cells (pDCs), T-helper cells, T-follicular helper cells (Tfhs), Th1 cells, Th2 cells, and tumor-infiltrating lymphocytes (TILs) were more active in the low-risk group, while macrophages were more active in the high-risk group (**Figure 7B**). In the gene set of immune-related pathways, clinical complete response (CCR), checkpoint, cytolytic activity, human leukocyte antigen (HLA), inflammation promoting, major histocompatibility complex (MHC) class I, T-cell co-inhibition, and T-cell co-stimulation were more active in the low-risk group than in the high-risk group (**Figure 7C**).

### 3.5 The role of prognostic model in immunotherapy

PD-1 and CTLA-4 are common target molecules for ICIs to exert antitumor effects. We compared the expression levels of PD-1 and CTLA-4 between different risk groups, and the results indicated that the expressions of PD-1 (**Figure 7D**, p< 0.001) and CTLA-4 (**Figure 7E**, p< 0.001) were significantly higher in the low-risk group than in the high-risk group. This suggested that patients in the low-risk group might benefit more from anti-PD-1 and anti-CTLA-4 therapy. The violin plots suggested that the treatment effect of the low-risk group was significantly better than that of the high-risk group, whether the treatment was anti-PD-1 alone (**Figure 7F**, p< 0.001), anti-CTLA-4 alone (**Figure 7G**, p< 0.001), or the combination of both (**Figure 7H**, p< 0.001), which was corroborated by the expression levels of PD-1 and CTLA-4 in the two groups.

## 4 Discussion

BRCA is the most common malignant tumor in women. In recent years, the incidence of BRCA has shown an obvious upward trend (20). Therefore, it is of great significance to search for biomarkers to predict the prognosis of patients with BRCA. Evidence showed that autophagy plays different roles in the occurrence and development of some malignant tumors, which can promote the occurrence and progress of tumors or inhibit tumors, depending on the nutritional status of the tumors at different stages and the influence of the TME and other factors (21). Wang et al. (22) found that exosomal miR-1910-3p promotes autophagy, proliferation, and metastasis of BRCA through the nuclear factor kappa B (NF-κB) signaling pathway. Tian et al. (23) confirmed that magnoflorine-induced autophagy improves the sensitivity of BRCA to doxorubicin through the protein kinase B (AKT)/mechanistic target of rapamycin (mTOR) and p38 signaling pathways. Chung et al. (24) proposed that adiponectin, C1Q, and collagen domain containing (ADIPOQ)/adiponectin, a cytokine, have the capability to induce autophagy in BRCA through the activation of AMP-activated protein kinase (AMPK)-Unc-51 like autophagy activating kinase 1 (ULK1) pathway mediated by serine/threonine kinase 11 (STK11, also known as LKB1). Studies have shown that autophagy-related genes (ARGs) and ARlncRNAs are closely related to the prognosis of BRCA (25, 26). Recently, with the rise in bioinformatics, there have been many prognostic models constructed based on ARGs (16) and ARlncRNAs (27) to predict the prognosis of patients with BRCA. However, we combined the ARlncRNAs prognostic model with tumor immunity for the first time in BRCA to explore the relationship between the two in detail. Thus, this model has the potential to guide the immunotherapy of patients with BRCA.

In this study, 10 ARlncRNAs significantly associated with prognosis were identified based on BRCA transcriptomic data and clinicopathological data in the TCGA database and ARGs in the HADb database. An ARlncRNA prognostic model was constructed based on these 10 ARlncRNAs. Multivariate cox regression analysis confirmed that the model could be used as an independent prognostic factor for BRCA. According to the median risk score, patients with BRCA were divided into the high- and low-risk groups, and the results showed that patients in the low-risk group had a better prognosis than those in the high-risk group. Subsequently, risk score, age, stage, T, N, and M were compared simultaneously to evaluate the predictive ability of the ARlncRNA prognostic model. The AUC value indicated that the predictive ability of the model was superior to other clinicopathological features. In addition, age, stage, T, N, and M of patients were evaluated using this model, which showed that age, stage, and N correlated significantly with the risk score, indicating that the model may be related to the progression of BRCA. Based on this study, we found that the ARlncRNA prognostic model is a clinically significant biomarker for BRCA. Among the 10 ARlncRNAs included in the model, seven (*AC090912.1*, *LINC01871*, *AL358472.3*, *AL122010.1*, *SEMA3B-AS1*, *BAIAP2-DT*, and *MAPT-AS1*) were considered to have a protective effect on the prognosis of BRCA. By contrast, three ARlncRNAs (*DNAH10OS*, *AC015819.1*, and *AC090198.1*) are risk factors for the prognosis of BRCA. Interestingly, six ARlncRNAs in the model, namely, *LINC01871* (27, 28), *AL122010.1* (28), *SEMA3B-AS*1 (29), *BAIAP2-DT* (27), *MAPT-AS1* (19, 30), and *AC090912.1* (19), have been reported in various prediction models of BRCA and are all protective biomarkers of BRCA, which is consistent with the result of this study. However, the rest in the model, namely, *AC015819.1*, *AC090198.1*, *DNAH10OS*, and *AL358472.3*, were reported for the first time in cancer, which suggests that a large number of lncRNAs have not been discovered yet and that lncRNAs have great potential as prognostic biomarkers for BRCA.

Next, to further explore the potential functions and pathways of the ARlncRNA model in BRCA, we performed GSEA enrichment analysis on the differentially expressed genes in the different risk groups. KEGG pathway enrichment analysis suggested that some pathways closely related to cancer were enriched in the high-risk group, while some immune-related pathways (antigen processing and presentation, natural-killer-cell-mediated cytotoxicity, and T-cell receptor signaling pathway), Janus kinase (JAK)-signal transducer and activator of transcription (STAT) signaling pathway, mitogen activated protein kinase (MAPK) signaling pathway, and the vascular endothelial growth factor (VEGF) signaling pathway were enriched in the low-risk group. Recently, studies have found that the JAK-STAT, MAPK, and VEGF signaling pathways are closely related to autophagy. For instance, Billah et al. (31) found that activation of interleukin-6-dependent JAK-STAT pathway upregulated the autophagy of cardiomyocytes, whereas inhibition of the JAK-STAT pathway had the opposite effect. Fan et al. (32) confirmed that activation of the reactive oxygen species (ROS)/MAPK signaling pathway induces autophagy in lung cancer cells. An et al. (33) discovered that autophagy regulates VEGF secretion in mesenchymal stem cells. Therefore, it is reasonable to speculate that autophagy may influence the occurrence and development of BRCA through the above-mentioned signaling pathways. In addition, GO functional enrichment analysis indicated that differentially expressed genes were enriched in a large number of immune-related functions. According to the results of KEGG enrichment analysis for ARGs co-expressed with 10 ARlncRNAs, we found that the ARGs were enriched in some pathways, such as some autophagy-related pathways, MAPK signaling pathways, and neurological lesion-related pathways. In conclusion, autophagy and tumor immunity play a vital role in the prognosis of BRCA.

Recently, a large amount of evidence has indicated that autophagy is closely related to tumor immunity. For example, Yamamoto et al. (34) found that inhibition of autophagy enhances anti-tumor immune activity and improves ICI efficacy in pancreatic cancer. Li et al. (35) confirmed that autophagy is associated with T-cell-mediated anti-tumor effects and sensitivity to anti-PD1/programmed cell death 1 ligand 1 (PDL1) drugs in TNBC. In addition, it has been widely recognized that the TME can influence the development and progression of cancers (36, 37). Therefore, we explored the relationship between the ARlncRNA prognosis model and the TME in detail using ssGSEA. We found that a number of immune cells and immune pathways were more active in the low-risk group, and most of them were involved in autophagy. For example, Ding et al. (38) found that β-glucan induces DC autophagy, which was beneficial to their maturation. He et al. (39) observed that CD36-mediated autophagy was closely related to humoral immunity in B cells. Li et al. (35) proved that autophagy plays a key role in TNBC resistance to T-cell-mediated cytotoxicity. El-Darawish et al. (40) found that IL-18-mediated autophagy regulates NK cell proliferation in mice. Zarogoulidis et al. (41) discovered that inhibition of autophagy can induce upregulation of CD4+ TILs. Li et al. (42) demonstrated that autophagy correlates strongly with central nervous system inflammation. Yamamoto et al. (34) revealed that autophagy-mediated MHC-I degradation plays an important role in immune evasion in pancreatic cancer. Interestingly, macrophage activity was higher in the high-risk group, possibly suggesting an association with poor prognosis. Studies have proven that M2 macrophages, representing tumor-associated macrophages, promotes the progression of BRCA *via* polarization (43). These results above indicated that autophagy probably influences the prognosis of BRCA *via* immune regulation.

Recently, anti-PD1 and anti-CTLA4 therapy have been suggested to be effective in BRCA (44). In this study, we analyzed the expression levels of PD1 and CTLA4 in the ARlncRNA model, and the results indicated that the expression levels of PD1 and CTLA4 were significantly increased in the low-risk group compared with those in the high-risk group, suggesting that ICIs probably have better efficacy in the low-risk group. Subsequently, these results were confirmed by ICI efficacy evaluation in the ARlncRNA model. We found that patients in the low-risk group experienced better efficacy whether they were treated with anti-PD1 alone, anti-CTLA4 alone, or a combination of both. In conclusion, the ARlncRNA model is expected to be a marker to guide treatment using ICIs in BRCA.

Although our study has certain clinical significance for judging the prognosis of patients with BRCA and guiding the individual use of ICIs in such patient, there are still some limitations. First, we only used data from TCGA database, lacking cross-validation from other databases. Second, the potential mechanisms of autophagy and immune factors that we identified as affecting the prognosis of BRCA still require experimental verification

## 5 Conclusion

We constructed a prognostic model based on 10 ARlncRNAs by performing comprehensive analysis of BRCA data. Compared with other clinicopathological characteristics, the ARlncRNA model provides a more reliable predictive ability. In addition, this model has certain value in guiding the individual use of ICIs in patients with BRCA. Therefore, the ARlncRNA model is expected to become an important biological indicator of BRCA.

## Data availability statement

The original contributions presented in the study are included in the article/**Supplementary Material**. Further inquiries can be directed to the corresponding author.

## Author contributions

JC and WW contributed to provide the idea for the study. XL, YXZ, SY, and JL contributed to the execution of the R language. JC was in charge of writing the paper. MW, JD, JY, YY, and YZ made a significant contribution to the discussion of the paper. All authors contributed to the article and approved the submitted version.
