# Supplementary material for: An autophagy-related long non-coding RNA prognostic model and related immune research for female breast cancer
Source: Front Oncol. 2022 Dec 15;12:929240. doi: 10.3389/fonc.2022.929240 (PMC9798206; doi:10.3389/fonc.2022.929240)
Supplement: Supplementary file 3 [file Image_1.pdf]

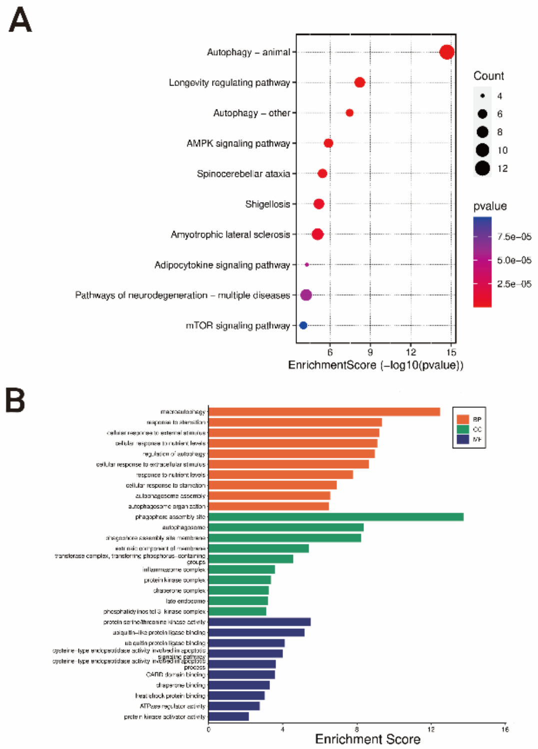

(A) KEGG pathway enrichment analysis indicating that several autophagy-related pathways were enriched. (B) GO enrichment analysis suggesting that several autophagy-related functions were enriched.
